# Supplementary material for: Deformation dynamics of nanopores upon water imbibition
Source: Proc Natl Acad Sci U S A. 2024 Sep 12;121(38):e2318386121. doi: 10.1073/pnas.2318386121 (PMC11420158; doi:10.1073/pnas.2318386121)
Supplement: Supplementary file 1 — Appendix 01 (PDF) [file pnas.2318386121.sapp.pdf]

Supporting Information for

Deformation Dynamics of Nanopores upon Water Imbibition

Juan Sanchez, Lars Dammann, Laura Gallardo, Zhuoqing Li, Michael Fröba, Robert H. Meißner, Howard A. Stone, Patrick Huber

To whom correspondence should be addressed. E-mail: patrick.huber@tuhh.de

This PDF file includes:

Supporting text
Figs. S1 to S2
Legends for Movies S1 to S3
SI References

Other supporting materials for this manuscript include the following:

Movies S1 to S3

Supporting Information Text

Methods

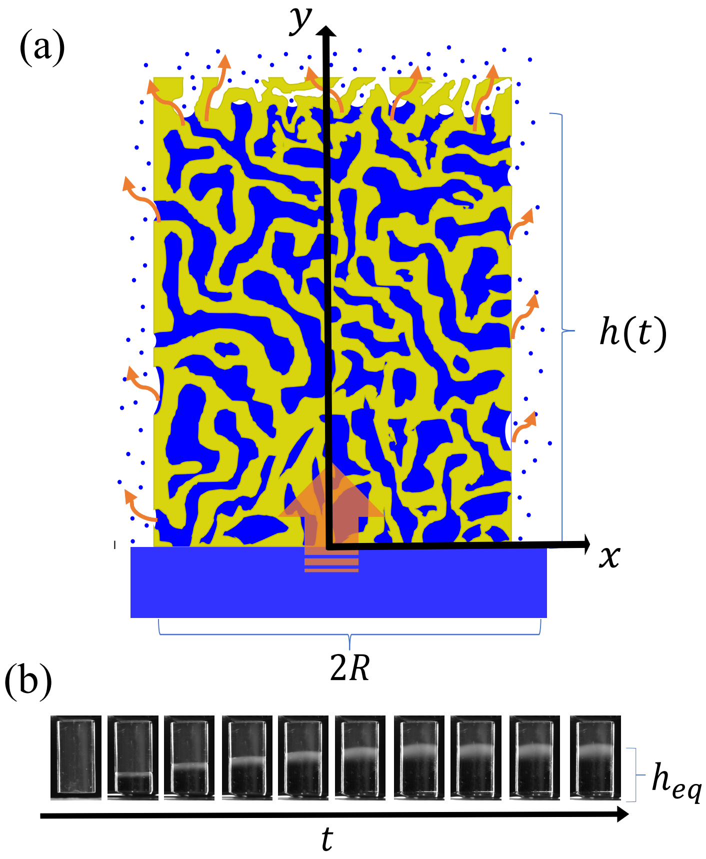

Fig. S1. Evaporation-Imbibition Equilibrium in Nanoporous Vycor Glass. (a) Schematic 2D cut of a cylindrical Vycor glass monolith aligned with its rotation axis parallel to the y direction, in contact with a water reservoir (blue rectangular area below). The radius of the cylinder is R and the mean position of the capillary rise front is denoted as $h(t)$. The big orange arrow indicates the main direction of the imbibition flow and the little orange arrows indicate the evaporation at the lateral surface. (b) Snapshots of a water imbibition experiment in nanoporous Vycor glass ($r_\mathrm{p} = 3.4\,\mathrm{nm}$, pre-imbibition humidity $RH \approx 5\%$) performed with an unsealed sample to allow evaporation as indicated in (a) (humidity during imbibition $RH \approx 50\%$). Snapshots are taken every hour. The arresting of the front is visible 6 hours after the start.

Derivation of capillary-rise dynamics during simultaneous evaporation loss. To solve the problem of the capillary rise including evaporation we begin by describing the infiltrated volume rate as the addition of the capillarity-driven flow (intake) and the volume loss due to evaporation.

$$\dot{V}_\mathrm{gain} = \dot{V}_\mathrm{imbi} + \dot{V}_\mathrm{evap} \quad [1]$$

We work with a cylindrical porous sample with radius R and cross section $A = 2R^2$ (see Fig. S1a). We assume a uniform imbibition profile and focus on the vertical flow (y direction). The slightly curved imbibition front visible in the snapshots in Fig. S1b shows that the vertical transport velocity decreases along the lateral x direction, being maximum at the center ($x = 0$) and minimum at $x = R$, due to the effect of evaporation through the lateral surface of the sample. As a first approximation, this effect is ignored, allowing us to treat the problem as one dimensional. We consider a simple evaporation model where the volume loss is proportional to the exposed wet surface, assuming a constant evaporation rate q quantified as volume of liquid water loss per surface area. Here q is a function of the humidity in the sample environment. The capillarity-driven flow of a liquid with dynamic viscosity flowing through a porous matrix with cross-sectional area A, initial porosity ϕ_i and permeability K, driven by a pressure drop Δp can be understood based on Darcy's law. We rewrite Eq. 1 in terms of the infiltrated length $h(t)$:

$$A \cdot \phi_\mathrm{i} \cdot \dot{h}(t) = A \cdot \frac{K}{\eta}\frac{\Delta p}{h} - q \cdot 2\pi R \cdot h(t), \quad [2]$$

or alternatively

$$\frac{dh(t)}{dt} = \frac{K\Delta p}{\eta \phi_\mathrm{i} h(t)} - \frac{2q}{R\phi_\mathrm{i}} \cdot h(t), \quad [3]$$

which is a first- order nonlinear ordinary differential equation (ODE). Multiplying Eq. 3 by $h(t)$ yields a linear ODE for $h(t)^2$. It can be immediately solved resulting:

$$h(t) = \sqrt{C_2 \cdot e^{-\frac{4q}{R\phi_\mathrm{i}}t} + \frac{K\Delta pR}{2q\eta}}, \qquad [4]$$

where the constant is set to $C_2 = -\frac{K\Delta pR}{2q\eta}$ by imposing the initial condition $h(t=0) = 0$. Introducing the characteristic time $t_\mathrm{E} = R\phi_\mathrm{i}/4q$ of the exponential decay term reveals that the steady state is reached for $t \gg t_\mathrm{E}$, i.e., for long times $h(t)$ tends to reach a saturation or equilibrium value $h_\mathrm{eq} = \sqrt{\frac{K\Delta pR}{2q\mu}}$. Note that every variable involved in h_eq is accessible for the case of water imbibition in Vycor glass (1). The solution to our problem can therefore be expressed as:

$$h(t) = h_\mathrm{eq} \cdot \sqrt{1 - e^{-\frac{4q}{R\phi_\mathrm{i}}t}}. \qquad [5]$$

Eq. 5 can be fit to our experimental data by variation of only two parameters: h_eq and q.

Moreover, a Taylor series analysis around $t = 0$ results in:

$$h(t) \simeq \; h_\mathrm{eq}\left(\left(\frac{t}{t_\mathrm{E}}\right)^{1/2} - \frac{1}{4}\left(\frac{t}{t_\mathrm{E}}\right)^{3/2} + \frac{5}{96}\left(\frac{t}{t_\mathrm{E}}\right)^{5/2}\right) + ..., \qquad [6]$$

which means that for imbibition times, $t \ll t_e = \frac{R\phi_\mathrm{i}}{4q}$, where higher orders of the Taylor series are negligible,

$$h(t) \approx \sqrt{\frac{4K\Delta p}{2\eta\phi_\mathrm{i}}}\sqrt{t}. \qquad [7]$$

By substituting $\Delta p = 2\gamma/(r_\mathrm{p} - d)$ and $K = \frac{1}{8}\frac{(r_\mathrm{p}-d)^4\phi_o}{r_\mathrm{p}^2\tau}$ as explained in (1), Eq. 7 transforms into:

$$h(t) = \sqrt{\frac{\gamma}{2\eta\tau\xi}\frac{(r_\mathrm{p} - d)^3}{r_\mathrm{p}^2}}\sqrt{t} \qquad [8]$$

which is our MLW law, already described in the main manuscript, and responsible for the imbibition-driven capillary rise dynamics. In other words, for short times $t \ll t_e = \frac{R\phi_\mathrm{i}}{4q}$, imbibition dominates over evaporation and the rising dynamics can be described exclusively in terms of the classical L-W square-root-of-time dependent law that states the competition between viscous drag and capillary pressure. Note that these findings agree with an analysis of wicking in paper-based microfluidic devices in the presence of evaporative flows (2)

Molecular Dynamics Imbibition Study.

Simulation Details. As a host material for the simulated imbibition the amorphous silica pore from Ref. (3, 4) consisting of 11610 individual atoms is used. However, the original host material is periodically repeated in every direction at simulation box edges. The periodic repetitions are required in lateral pore direction, but need to be broken in the longitudinal direction of the pore to connect the pore ends to the water reservoirs. Discontinuing the pore at the simulation box edges resulted in dangling silica atoms at the two created pore ends. The dangling silicon atoms have been removed. Subsequently, dangling oxygen atoms have then been saturated with hydrogen atoms.

During all simulations the hydrogen atoms in the silica system and the water molecules are constrained by the SHAKE algorithm (5). Furthermore, a Particle–Particle Particle–Mesh ($\mathrm{P^3M}$) solver (6) is used to calculate long range Coulombic interactions. All thermostats and barostats have been employed with a damping of 100 fs and 1000 fs respectively throughout every part of the simulation.

During the initial part of the Molecular Dynamics imbibition study it is important to differentiate between physically plausible and non-physical water and strain dynamics. Non-physical dynamics may originate from the sudden appearing interactions of the newly created water reservoir with the nanoporous system. To that end the imbibition is prepared by adding a water reservoir of 22680 molecules at the longitudinal end of the porous matrix. Due to periodic boundary conditions the water in the reservoir can freely transition from one end of the simulation box to the other. To relax the system in respect to the new interaction of the water reservoir with the nanoporous system an isothermal-isobaric ensemble (NpT) is simulated for a total of 25 ps. To prevent a penetration of the water column into the porous system during the relaxation phase the water molecules that penetrated deeper into the porous matrix then 5 Å are deleted every 10 fs. The 5 Å are measured from the position of the Si atoms being closest to the longitudinal end of the pore. This way the porous matrix can equilibrate to the water contact while no imbibition into the pores occurs. Additionally no water menisci can form in the pores. The equilibrated system was used as a starting point for each of the 20 simulations. To make the individual simulations independent in its trajectories an additional isothermal-isochoric (NVT) ensemble has been computed for a different amount of time for each simulations, e.g. 20 fs, 40 fs, ..., 400 fs, where only water molecule positions have been integrated. Water molecule positions of a thin layer of 3 Å into the porous system have not been integrated during the simulation to act as a wall preventing the water molecules from entering the pores.

Measurement of Simulation Parameters. The water penetration length $h(t)$ is acquired from the simulation box extension along the longitudinal pore axis $L_z(t)$. Due to the 1 bar pressure the simulation box shrinks proportional to the amount of water that transitioned from the water reservoir into the pore. The total distance the water can penetrate into the pore is measured by the smallest distance h_{pore} of water molecules originating from the water reservoirs across the longitudinal pore space devoid of water in the initial system configuration. The penetration length of one water column can be calculated from the simulation box extension through $h(t) = \frac{1}{2}\frac{[L_z^{\text{final}} - L_z(t)]h_{\text{pore}}}{L_z^{\text{final}}(t) - L_z^0}$, where L_z^0 describes the initial simulation box extension and L_z^{final} the final one.

The lateral pore strain can be indirectly measured through the simulation box extensions along the lateral pore direction. Since the simulation box extension is coupled in the lateral dimensions the box dimension in x direction $L_{\text{x}}(t)$ or y direction $L_y(t)$ will yield the same results in terms of strain. The lateral pore strain is calculated by $\epsilon_\perp(t) = \frac{L_y(t) - L_y^0}{L_y^0}$ with L_y^0 being the initial box extension.

To measure the longitudinal pore extension all Si and bulk O atoms (that are the atoms not bound in a hydroxyl OH surface group) in the already wetted part at the initial configuration of the simulation study are considered. That are precisely the atoms situated before the 5 Å into the pores after that water molecules are deleted every 10 fs during the relaxation phase. The two groups of considered bulk atoms contain 228 atoms on one pore side and 217 on the other. To calculate the strain in the porous material for every time step every distance r_{ij} between every atom in the two bulk atom groups is calculated. For each time step this yields $n = 49476$ distances. From those distances the z_{ij} component is used to calculate the longitudinal strain for every time step. The mean strain of all distances is then used as longitudinal strain $\epsilon_\parallel(t) = 1/n \sum_{ij} \frac{z_{ij}(t) - z_{ij}^0}{z_{ij}^0}$ with z_{ij}^0 being the z components of the distances at time step 0.

Inertia Governed Imbibition Regime. The simulated imbibition dynamics display an initial linear regime $h(t) = v \cdot t$ for approximately 3 ps. This regime can be explained by an imbibition process governed solely by inertia of the imbibing water (7, 8). The theoretical expected velocity of the inertia governed regime can be calculated by considering that the influence of the water viscosity is negligible for initial imbibition times. The driving force F in a laminar Hagen-Poiseuille flow is $F = 2\pi r\gamma$. The instantaneous momentum p of the liquid equates to $p = \pi r^2 \rho h \frac{dh}{dt}$. Therefore

$$\frac{dp}{dt} = F \quad \Rightarrow \quad \frac{d}{dt}\left[\pi r^2 \rho h(t) \frac{dh(t)}{dt}\right] = 2\pi r\gamma \quad \Rightarrow \quad \frac{d}{dt}\left[h(t)\frac{dh(t)}{dt}\right] = \frac{2\gamma}{\rho r}$$

$$\Rightarrow \quad \frac{dh(t)^2}{dt^2} = \frac{4\gamma}{\rho r} \quad \Rightarrow \quad h(t)^2 = \frac{2\gamma}{\rho r}t^2 \quad \Rightarrow \quad h(t) = \underbrace{\sqrt{\frac{2\gamma}{\rho r}}}_{v}\, t.$$

With the pore radius of $r \approx 1.5$ nm, the surface tension of $\gamma = 72\,\text{mN}\,\text{m}^{-1}$ (9), and the water density of $\rho = 1000\,\text{kg}\,\text{m}^{-3}$ the theoretical derived water column velocity calculates to $v_{\text{theo}} \approx 3.1 \times 10^{-3}\,\text{nm}\,\text{ps}^{-1}$. Fitting a linear function to the initial simulated imbibition results in a velocity of $v = 1.1 \times 10^{-3}\,\text{nm}\,\text{ps}^{-1}$ (see Fig. S2). Thus, the simulated value derived from the simulation is a factor of 3 smaller than expected from theoretical considerations. This deviation is consistent with measurements of Quéré (8) of the linear imbibition regime for macroscopic capillaries, where the deviation was explained by consideration of a dynamic contact angle θ at the liquid front. Taking the influence of the dynamic contact angle into consideration the imbibition velocity has to be altered by a factor $\sqrt{\cos\theta}$. For the theoretical imbibition velocity in the linear regime to agree with the simulated imbibition the contact angle in the inertia dominated regime has to be of the order of $\theta = 83°$ in the simulations. Following theoretical considerations the transition from the inertia dominated regime to the viscosity dominated regime should occur at the time $\tau = \rho r^2/4\eta \approx 0.7$ ps. However, as visible in Fig. S2 during the simulation the transition occurs at around 3.2 ps. Analog to the discrepancy in velocity the difference in regime transition time could be potentially explained by a dynamically increased contact angle $\theta = 83°$ leading to a significantly reduced initial imbibition speed. Indeed, a visual inspection of the first two simulation snapshots shown in Fig. 4 of the main manuscript corroborates this conclusion.

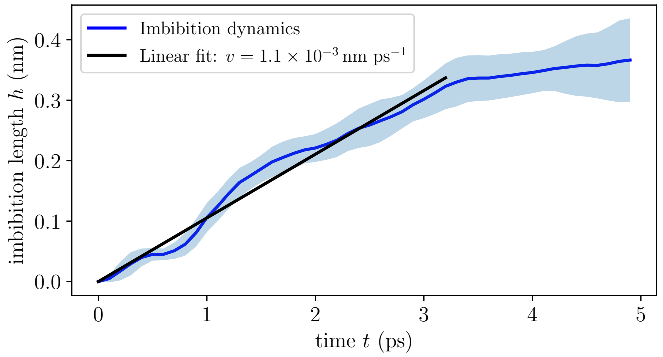

Fig. S2. Initial linear regime of the imbibition dynamics as revealed by Molecular Dynamics simulation. The measured water inertia governed imbibition velocity is $v = 1.1 \times 10^{-3}\,\mathrm{nm\,ps^{-1}}$ which is a factor 3 smaller than the theoretically estimated $v_{\mathrm{theo}} = 3.1 \times 10^{-3}\,\mathrm{nm\,ps^{-1}}$. The shaded area represents the standard deviation of the imbibitions across the simulation ensembles.

Movie S1. Video of our optical imaging experiment on water imbibition in nanoporous Vycor glass, in absence of evaporation. The sample length is $\sim 12\,\mathrm{mm}$, and the side and top of the sample are sealed using transparent adhesive film (TESA film®). The real time duration of the experiment is 2.05 hours.

Movie S2. Video of our optical imaging experiment on water imbibition in nanoporous Vycor glass, with evaporation. The sample length is $\sim 19\,\mathrm{mm}$, and the side and top of the sample are not sealed allowing evaporation. The real time duration of the experiment is 27.87 hours.

Movie S3. Video of snapshots originating from the first simulated $780\,\mathrm{ps}$ (regime I) of one exemplary imbibition simulation trajectory. The simulation ensemble used to investigate the imbibition dynamics and the imbibition-induced strain consists of 20 trajectories with independent starting conditions.

References

1. S Gruener, T Hofmann, D Wallacher, AV Kityk, P Huber, Capillary rise of water in hydrophilic nanopores. *Phys. Rev. E - Stat. Nonlinear, Soft Matter Phys.* **79**, 067301 (2009).
2. CK Camplisson, KM Schilling, WL Pedrotti, HA Stone, AW Martinez, Two-ply channels for faster wicking in paper-based microfluidic devices. *Lab on a Chip* **15**, 4461–4466 (2015).
3. P Ugliengo, et al., Realistic Models of Hydroxylated Amorphous Silica Surfaces and MCM-41 Mesoporous Material Simulated by Large-scale Periodic B3LYP Calculations. *Adv. Mater.* **20**, 4579–4583 (2008).
4. M Delle Piane, M Corno, A Pedone, R Dovesi, P Ugliengo, Large-scale B3LYP simulations of ibuprofen adsorbed in MCM-41 mesoporous silica as drug delivery system. *J. Phys. Chem. C* **118**, 26737–26749 (2014).
5. JP Ryckaert, G Ciccotti, HJ Berendsen, Numerical integration of the cartesian equations of motion of a system with constraints: molecular dynamics of n-alkanes. *J. Comput. Phys.* **23**, 327–341 (1977).
6. RW Hockney, JW Eastwood, The particle-mesh force calculation. *Comput. Simul. Using Part. Adam Hilger, Bristol New York, NY, USA* pp. 120–165 (1989).
7. C Bosanquet, LV. On the flow of liquids into capillary tubes . *The London, Edinburgh, Dublin Philos. Mag. J. Sci.* **45**, 525–531 (1923).
8. D Quere, Inertial capillarity. *Eur. Lett.* **39**, 533 (1997).
9. J Alejandre, GA Chapela, The surface tension of TIP4P/2005 water model using the Ewald sums for the dispersion interactions. *J. Chem. Phys.* **132**, 926 (2010).
